# A Pragmatic Application of the RE-AIM Framework for Evaluating the Implementation of Physical Activity as a Standard of Care in Health Systems

**DOI:** 10.5888/pcd15.170344

**Published:** 2018-05-10

**Authors:** Mark Stoutenberg, Karla I. Galaviz, Felipe Lobelo, Elizabeth Joy, Gregory W. Heath, Adrian Hutber, Paul Estabrooks

**Affiliations:** 1 Department of Public Health Sciences, University of Miami, Miami, Florida; 2 Hubert Department of Global Health, Rollins School of Public Health, Emory University, Atlanta, Georgia; 3 Exercise is Medicine Global Research and Collaboration Center; Atlanta, Georgia; 4 Community Health & Food and Nutrition, Intermountain Healthcare, Salt Lake City, Utah; 5 Department of Health and Human Performance, University of Tennessee Chattanooga, Chattanooga, Tennessee; 6 Exercise is Medicine, American College of Sports Medicine, Indianapolis, Indiana; 7 Department of Health Promotion, Social and Behavioral Health, University of Nebraska Medical Center, Omaha, Nebraska

## Abstract

**Introduction:**

Exercise is Medicine (EIM) is an initiative that seeks to integrate physical activity assessment, prescription, and patient referral as a standard in patient care. Methods to assess this integration have lagged behind its implementation.

**Purpose and Objectives:**

The purpose of this work is to provide a pragmatic framework to guide health care systems in assessing the implementation and impact of EIM.

**Evaluation Methods:**

A working group of experts from health care, public health, and implementation science convened to develop an evaluation model based on the RE-AIM (Reach, Effectiveness, Adoption, Implementation, and Maintenance) framework. The working group aimed to provide pragmatic guidance on operationalizing EIM across the different RE-AIM dimensions based on data typically available in health care settings.

**Results:**

The Reach of EIM can be determined by the number and proportion of patients that were screened for physical inactivity, received brief counseling and/or a physical activity prescription, and were referred to physical activity resources. Effectiveness can be assessed through self-reported changes in physical activity, cardiometabolic biometric factors, incidence/burden of chronic disease, as well as health care utilization and costs. Adoption includes assessing the number and representativeness of health care settings that adopt any component of EIM, and Implementation involves assessing the extent to which health care teams implement EIM in their clinic. Finally, Maintenance involves assessing the long-term effectiveness (patient level) and sustained implementation (clinic level) of EIM in a given health care setting.

**Implications for Public Health:**

The availability of a standardized, pragmatic, evaluation framework is critical in determining the impact of implementing EIM as a standard of care across health care systems.

## Introduction

Physical activity prevents, delays, or is used to manage many chronic conditions, such as diabetes, high blood pressure, and cardiovascular diseases (1,2). Yet, in 2014, only 21.5% of American adults met both aerobic and muscle strengthening guidelines (3). Although this percentage reflects an increase from 15.1% in 2000, these physical activity levels are still well below the recommended levels necessary to achieve population health benefits. Insufficient physical activity is estimated to account for 11.1% of aggregated health care expenditures in the United States, which translates to $117 billion, or slightly more than $1,300 per capita for inactive, versus active, persons (4).

Numerous reports have advocated for a collaborative approach to improving physical activity levels across multiple sectors of society (5,6). Given that more than 75% of all US adults had contact with a health care professional from 2013 to 2015 (7), multiple calls to action have advocated for the health care sector to take on a greater role in promoting physical activity at a population level (8–10). In 2014, the US Preventive Services Task Force recommended that adults with cardiovascular disease risk factors should be referred to intensive behavioral counseling interventions to promote physical activity and healthful diet (11).

During the past several decades, several studies have evaluated single-level interventions to integrate physical activity into health care settings, such as physician counseling (12,13), assessing patient physical activity levels (14,15), and providing patients with a physical activity prescription (16,17). A small number of multilevel interventions have been conducted in health care settings to support physicians in their physical activity counseling efforts (18,19). Meta-analyses and systematic reviews show that physician counseling and exercise referral systems lead to improvements in patient physical activity levels for up to 12 months (20,21). Furthermore, physical activity counseling and referral schemes can provide early return on investment because of lower health care utilization and costs (22,23). However, few efforts have evaluated the potential for large-scale implementation of these referral schemes in clinical practices across larger health care systems.

Simultaneously, we are witnessing a rapid transformation in clinical practice within health systems with the goal of achieving the new “quintuple” aim of health care: improving the health of populations, improving the patient experience, increasing patient engagement, reducing the per capita cost of health care, and improving the work–life balance of health care providers (24). Integral to achieving these aims are strategies that 1) aggregate and analyze patient data, 2) identify at-risk patient groups, 3) develop risk-specific action plans, and 4) create patient-engagement tools (25). To incorporate these strategies in a systematic approach to integrating physical activity into health systems, Exercise is Medicine (EIM) was launched by the American College of Sports Medicine (ACSM) in 2007. The goal of EIM is to make physical activity a standard in patient health care for the prevention and treatment of chronic diseases (26). To date, EIM has been adopted in more than 40 countries worldwide (27), as well as in clinic settings (28) and entire health care systems (29) in the United States.

## Purpose and Objectives 

Despite greater reliance on data-driven approaches to patient health, methods to assess the integration of physical activity into typical health care practices — across both patient and organizational indicators — has lagged behind its implementation. The complex processes and multilevel factors associated with implementation need to be formally assessed to inform future efforts. Developing data collection and evaluation strategies that extend beyond traditional markers of efficacy or effectiveness is critical in providing more generalizable evidence for physical activity initiatives in health care settings (30). At the same time, it is essential to develop *pragmatic* evaluation strategies that take into consideration the barriers experienced by health care providers (31), that account for the realities and constraints of the current health care environment, and that are feasible in real-world settings (32).

Our understanding of the implementation of physical activity interventions in health care settings is limited by a lack of comprehensive evaluation frameworks. Recognizing this limitation, ACSM convened a working group to develop a pragmatic framework for evaluating the implementation of EIM as a standard of care in health systems that can be used by researchers, clinicians, and policy makers around the world.

## Evaluation Methods

**Figure 1:**
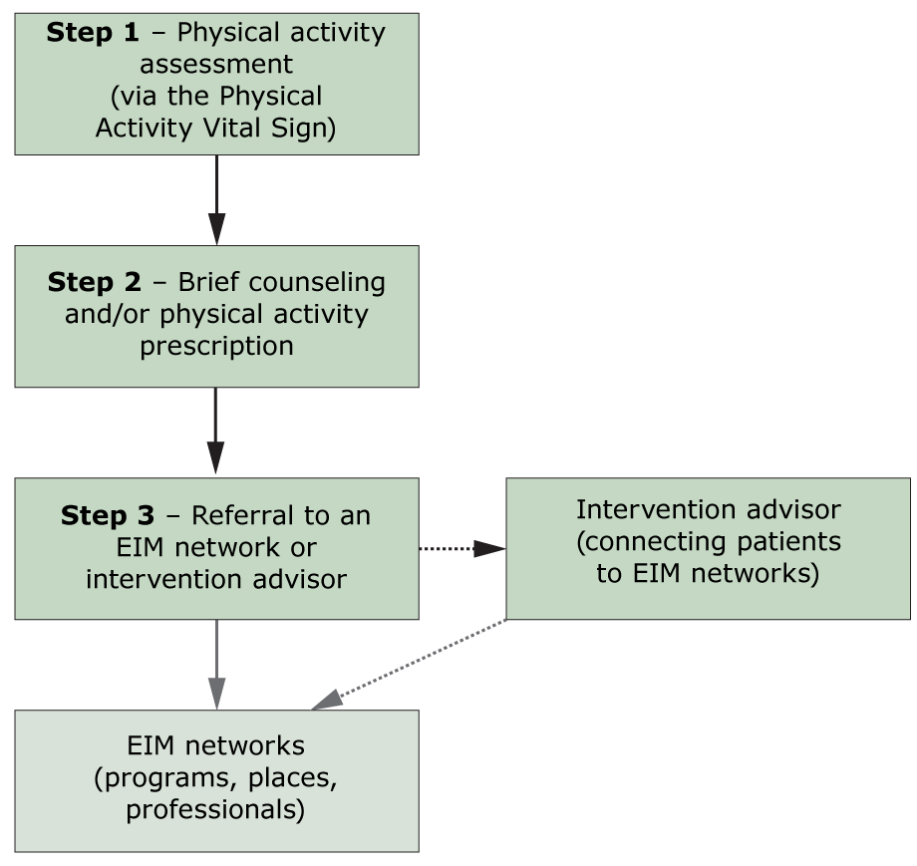
The clinical phase of the EIM Solution. Steps 1 through 3 of the EIM Solution take place primarily in the clinic setting and involve patients having their physical activity levels assessed, receiving brief counseling from a member of the health care team and/or receiving a semi-customized physical activity prescription, and receiving a referral to EIM network of physical activity resources. In some health systems, patients may also interact with, or be referred to, an intervention advisor. The role of the intervention advisor could include behavior change counseling and connecting the patient to the EIM network of physical activity resources. Dashed lines indicate an indirect or alternative pathway. The gray box indicates the community phase of the EIM Solution. Abbreviation: EIM, Exercise is Medicine.

A working group of 7 experts from health care, public health, family and sports medicine, and implementation science, convened to develop a model for evaluating the implementation of the EIM Solution in health care systems. The EIM Solution is the strategic implementation of physical activity in health care settings that involves a series of discrete steps designed to create clinical–community linkages to engage patients in sustained physical activity (26). In the clinic setting, the first 3 steps of the EIM Solution include 1) systematically *assessing* and recording patient physical activity levels, 2) providing patients with brief physical activity counseling and/or a physical activity *prescription*, and 3) *referring* patients to a network of physical activity resources for guidance and support (Figure 1). In some health systems, an intervention advisor, a role filled by a person in the health system, such as a care manager, nurse practitioner, or a health coach, is necessary to facilitate the referral process by providing basic behavioral counseling and connecting patients to appropriate physical activity resources.

When identifying potentially eligible patients to engage in the EIM Solution, health systems adopting the EIM Solution should aim to assess the physical activity levels of most of their patients (step 1). Assessing physical activity levels should be conducted as if it were a vital sign (8) in the electronic medical record (EMR), similar to body weight or blood pressure, with all patients in the health system, except for patients for whom it is clearly not relevant (ie, patients with acute illness). It is recommended that the Physical Activity Vital Sign (PAVS), a brief, pragmatic assessment tool that has been tested and validated in several health systems (33,34), be used to capture data on patient physical activity levels to standardize measures across different health systems. The PAVS can be administered, typically in 30-seconds or less, by any member of the health care team, and has good face validity in identifying people not meeting national physical activity guidelines (35). People not engaging in 150 minutes of moderate-intensity physical activity per week are then eligible to receive a physical activity prescription and/or brief counseling (step 2), followed by a referral to a network of physical activity resources (step 3).

**Figure 2:**
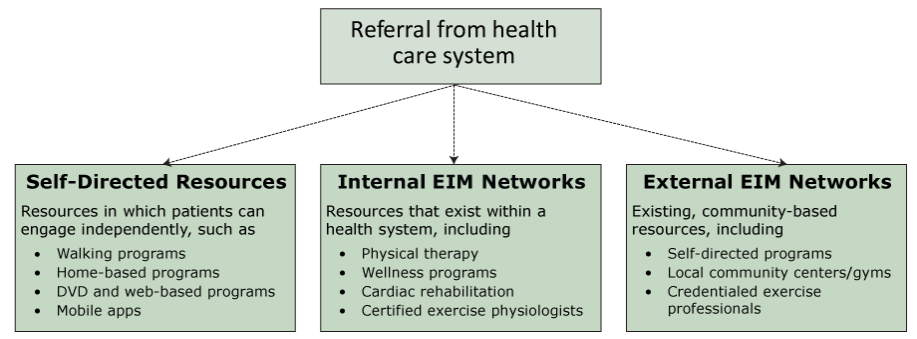
The “community” phase of the EIM Solution. The fourth step of the EIM Solution involves the linkage of patients from health care systems to a supportive network of physical activity programs, places, and professionals. These EIM networks may be developed by using existing programs and professionals internally within a health system or externally in the community setting or connecting patients to resources for self-directed management. The dashed lines indicate alternative pathways. Abbreviation: EIM, Exercise is Medicine. [A text version of this figure is also available.]

The fourth step of the EIM Solution involves the development of EIM networks consisting of physical activity programs, places and professionals capable of receiving patients referred from health care providers (Figure 2). EIM networks may include 1) self-directed, b) internal (ie, within the health system), or c) external (ie, community-based) physical activity resources. Self-directed resources include internet-based programs or smart-phone apps that support patient autonomy in becoming more physically active. Internal resources include physical therapists, wellness programs and facilities, and rehabilitation programs available to patients in a health system. An internal network may be a compilation of existing resources or comprise a more formal, standardized process to ensure a consistent level of quality and performance. Internal EIM networks will likely not have sufficient capacity to accommodate all referred patients. Therefore, referrals will also need to link patients to external resources located in the community, such as local places (eg, YMCAs), evidence-based programs, and credentialed exercise professionals (36). For quality control purposes, the programs, places and professionals in an external network may be required to meet established standards to receive patients from a health care system.

The working group first met in-person in January 2016 to develop an outline for the evaluation framework. Further communications refined the framework, which was presented at the 2016 ACSM annual meeting. The goals of the working group were to provide 1) pragmatic guidance on operationalizing and evaluating the implementation of the EIM Solution using data that is typically available in health care settings, and 2) recommendations for assessing additional indicators where existing data may not be readily available. The working group also identified indicators for the community settings (step 4 of the EIM Solution) to evaluate patient engagement and participation in internal and external EIM networks.

The RE-AIM (Reach, Effectiveness, Adoption, Implementation, and Maintenance) framework informed the development of the evaluation model. RE-AIM was selected because of its ability to provide an approach to planning and evaluation that balances factors related to both internal and external validity while focusing on patient and organization level outcomes. At the patient level, RE-AIM assesses the degree to which EIM reaches a large and representative proportion of at-risk (ie, physically inactive) patients and effectively produces and maintains changes in their health. At the organizational level, RE-AIM determines the ease and degree to which EIM is adopted by health systems, implemented with high fidelity, and sustained long-term (37). RE-AIM has been used in evaluating diabetes prevention and weight loss programs, and nutrition interventions (38,39).

## Results

A consensus was reached by the working group for the development of an evaluation model that relies on readily available data that are collected as a part of routine clinical practice. The model provides guidance for an array of health systems — from smaller practices to large, clinically integrated health systems or networks — while allowing for basic comparisons across these different settings. The working group considered measures to be pragmatic if they could be collected as a part of standard practice, are inexpensive, actionable, placed a low burden on staff, and are sensitive to change over time (32). Given the availability of pragmatic data in health systems via EMRs, the working group focused on the clinical care components of the EIM Solution (steps 1–3), rather than patient engagement and participation (step 4), where pragmatic data are often not readily accessible to a health care system and/or evaluation team. The working group also provided recommendations for additional (or expanded) indicators that could be reasonably collected and assessed where existing health system data are not currently available.

### Evaluating the EIM Solution in clinical care settings

#### Reach

Reach can be assessed by estimating the number of patients that were 1) screened for their current physical activity levels, 2) received brief counseling and/or a physical activity prescription, and 3) were referred to physical activity programming. The proportion of participants reached can be estimated by dividing the number of patients receiving each of these steps over the pool of potentially eligible patients. Those eligible to receive physical activity counseling and/or a physical activity prescription (step 2) and a referral to physical activity programming (step 3) include patients not meeting national aerobic activity recommendations based on their physical activity assessment. The representativeness of patients engaged is determined by comparing characteristics (ie, age, body mass index [BMI], race/ethnicity, payer status) of those reached (numerator) to all eligible patients (denominator) for each of the first 3 steps of the EIM Solution.

For health care organizations that use an internal or external EIM network (step 4), an expanded reach indicator is used to collect information on the number and proportion of referred patients that participate in the EIM networks. Representativeness is determined by comparing the characteristics of patients who receive a referral and participate in an EIM network compared with 1) eligible patients who did not receive a referral and 2) eligible patients who received a referral but did not attend a physical activity program.

In most cases, data for assessing the reach of the EIM Solution should be available through the patient EMR. Notes in the EMR (ie, drop down menu options or manually entered notes) can be used to record physical activity counseling, provision of physical activity prescriptions, and referral to physical activity resources. For systems that do not have EMRs, health care teams can record results from the physical activity assessment, as well as notes for brief counseling and provision of physical activity prescriptions and referrals, on paper-based patient records.

#### Effectiveness

The effectiveness of integrating the EIM Solution into health care systems should be evaluated across each of the 3 clinic-based steps. Measures of effectiveness should include changes in 1) self-reported physical activity or, as technology advances, physical activity objectively assessed by wearable devices, 2) cardiometabolic biometric values, and 3) the incidence of chronic disease, disease burden, and/or disease complications. The impact of physical activity assessment, providing brief counseling and/or physical activity prescriptions, and giving physical activity referrals on each of these outcomes can be compared with patients that did not receive any of these steps.

Data for assessing the effectiveness of the EIM Solution should be available through the EMR and include data on patient physical activity levels and cardiometabolic biometric values, such as body weight, BMI, waist circumference, systolic and diastolic blood pressure, lipid concentrations, triglyceride levels, and fasting blood glucose levels. Data on disease incidence, burden, and complications may include disease rates (ie, diabetes, cardiovascular diseases) and chronic disease complications (ie, the Charlson Comorbidity Index) calculated from existing tools and/or captured in the EMR. Data from paper-based health records can also be used when EMR data are not available.

An expanded measure of effectiveness is to assess differences in health care utilization and costs between patients exposed, versus those not exposed, to any of the EIM Solution steps. Health care utilization and costs, such as the number of annual physician or emergency department visits, are considered expanded measures because these data may not be readily obtained from the EMR. A second expanded measure is to examine the dose response, based on whether a patient receives 1, 2, or all 3 steps of the EIM Solution, and measures of effectiveness. Similarly, the frequency with which patients receive steps of the EIM Solution over a defined period of time (ie, all visits within a calendar year) and how frequency affects patient outcomes (listed above) can also be examined.

#### Adoption

The adoption of the EIM Solution can be assessed at both the health care system and provider levels. At the system level, adoption includes the number and proportion of health settings that adopt any or all of the steps of the EIM Solution. Representativeness can be determined by comparing characteristics of the health settings (ie, number of providers, payer-mix ratio, support-staff-to-provider ratio) that adopt components of the EIM Solution with all other health settings that had the opportunity, but did not adopt the EIM Solution. Similarly, adoption can be assessed by determining the number, proportion and representativeness (ie, sex, age, specialty) of health care providers that adopt any of the clinical steps of the EIM Solution in their practices compared with peers in the same health setting that do not adopt the EIM Solution. A final adoption indicator is to characterize the extent to which EIM internal/external networks are developed by a clinic for use with their patients.

#### Implementation

Implementation assesses the extent to which the EIM Solution is carried out as intended in the clinic setting. Implementation can be assessed by determining the extent to which all 3 steps are conducted with each eligible patient (ie, the 3 steps of the EIM Solution are delivered to 40% of eligible patients). The level of implementation can also be examined as the extent, or proportion, to which health care providers implement 1, 2, or all 3 clinic-based components of the EIM Solution with their eligible patients. Characteristics of health care providers that implement the EIM Solution with a high proportion of their patients can be compared with those that implement it with a low proportion of their patients. Baseline implementation levels can serve as benchmarks (ie, health care providers delivered all 3 steps of the EIM Solution to 50% of their eligible patients) so that incremental goals for improvement can be adjusted over time. Expanded assessments of implementation can be obtained through surveying patients to determine the number, proportion, and representativeness of those who receive 1, 2, or all 3 of the clinic-based steps of the EIM Solution. Finally, the use of checklists by a member of the health care team or an evaluation team can be used to ensure fidelity in the delivery of the EIM Solution.

#### Maintenance

Indicators of maintenance should be assessed at both the patient and organizational level. At the patient level, maintenance includes the effects of physical activity assessment, counseling and/or prescription, and referral on long-term (6, 12, 24, 36 months) patient outcomes. Patient physical activity levels and other effectiveness outcomes over time can be compared with eligible patients that did not receive the EIM Solution. At the institutional level, indicators of maintenance include the long-term institutionalization and sustained delivery (6, 12, 24, 36 months) of the EIM Solution. This can be assessed by examining the rate of using the EIM Solution by health care teams over time. An expanded indicator would be to examine patient maintenance (ie, long-term physical activity levels) by the dose of the EIM Solution (ie, number of times physical activity levels were assessed) received.

### Evaluating physical activity referrals to internal and external EIM networks

The fourth step of the EIM Solution is the development and utilization of an EIM network consisting of physical activity resources located either internally within a health system or externally in the community. Evaluating the utilization of EIM networks in community settings poses a unique set of challenges because of a lack of integration with health systems. This lack of integration makes the transfer of patient information from one setting to another (ie, participation rates in community programs integrated into EMRs) difficult. Many of the implementation indicators for the utilization of EIM networks relies upon this integration and, therefore, are not considered a part of the pragmatic framework, but as part of our expanded model.

When examining referrals to an EIM network, the number and proportion of referred patients that interact with either an intervention advisor or an exercise professional or attend a physical activity program in the EIM network should be quantified. The referral success rate will quantify the number of patients participating in at least 1 session (numerator) over all patients referred to an EIM network (denominator). Characteristics of participating patients can be compared with 1) patients who did not receive a referral or 2) patients who received a referral but did not attend. An expanded reach indicator is to compare the number and representativeness of patients who attend 25%, 50%, and 75% of planned sessions with those who attend a lesser number, or none, of the sessions.

For effectiveness, outcomes for patients receiving counseling by intervention advisors, working with exercise professionals, or attending programs in an EIM network can be compared with referred patients who do not attend, or attend a fewer number of, sessions. To optimally assess both reach and effectiveness, summary data for attendance at programs in an EIM network need to be captured and available for analysis. This may be achieved by automatically migrating data on patient attendance to EMR files or through third-party software solutions. An expanded effectiveness indicator involves assessing the dose–response relationship of the number of physical activity sessions attended compared with improvements in patient outcomes.

The number of physical activity resources (ie, intervention advisors, programs, places, and professionals) that participate in an EIM network can serve as an indicator of adoption. Describing these resources available in an EIM network and their capacity to provide physical activity opportunities for referred patients is an essential component of adoption. An expanded adoption indicator is to examine the proportion and characteristics (ie, size of programs, target population) of physical activity resources that participate in an EIM network compared with 1) existing programs and professionals that were approached but did not participate and 2) all existing programs and professionals in a community regardless of whether or not they were approached to participate in the network. By considering all programs in a community, stakeholders will get a true indication of adoption rates and the level of penetration of the EIM network.

All measures of implementation for using EIM networks are considered expanded measures because these data are often not routinely collected. A first recommended implementation indicator is to examine the extent to which intervention advisors adhere to their training protocol and procedures in guiding patients to physical activity resources. Other implementation indicators include the extent to which exercise professionals adhere to training protocols in offering the physical activity programs as originally designed. Implementation measures, as described above, can be evaluated via checklists that monitor (ie, through direct observation) the fidelity with which intervention protocols are implemented by intervention advisors and exercise professionals. Finally, the costs to the physical activity programs, places, and professionals to participate in an EIM network and offer programming to patients should be recorded on an ongoing basis.

Maintenance of EIM networks should be examined at both the patient and the organizational level. The long-term (6, 12, 24, and 36 months) effects of referring patients to an intervention advisor, or directly to an EIM network, can be assessed by examining changes in physical activity levels and health outcomes compared with baseline levels. These long-term changes can be compared with patients who did not interact with an intervention advisor or participate in an EIM network, either by choice or because they did not receive a referral. This information can be obtained via review of patient data from their EMR or notes in paper-based records, combined with information on their participation in the EIM network. At the organizational level, the continuity of maintaining updated internal and external resources in the EIM network, as well as the length of time, number of programs and professionals, and the sustained delivery of programs and professionals in an EIM network should be assessed over time.

### Costs of implementing the EIM Solution

A final component of the evaluation framework is to evaluate the costs of implementing the various components of the EIM Solution. Data on costs should be captured for each indicator in the RE-AIM framework. First, as a part of evaluating the effectiveness of the EIM Solution, data on changes in health care utilization costs, as well as laboratory and prescription drug expenditures, should be captured through insurance and billing charges. Second, the costs of adopting methods to 1) integrate physical activity assessment into the EMR, 2) provide patients with physical activity counseling and/or prescriptions, and 3) provide physical activity referrals should be collected. These adoption costs will typically appear as 1-time fixed expenditures. When evaluating the costs to implement the EIM Solution, it is necessary to track personnel costs, such as the training of providers and the time that they spend implementing the EIM Solution. Costs associated with personnel time spent implementing the EIM Solution in a clinic setting is an indirect process that is difficult, but necessary, to quantify. Long-term costs include expenses associated with maintaining the systems that support the EIM Solution in a health system (ie, updating software systems and programs). Finally, an overlooked expense includes the funds necessary to provide ongoing evaluation (ie, data extraction and analytics) of the EIM Solution.

For step 4 of the EIM Solution it is important to track the costs associated with developing and maintaining EIM networks, such as ongoing staff hiring and training. In internal EIM networks, data on these costs will be available through the health system and their accounting records. In external EIM networks, the costs of physical activity programs, places, and professionals participating in an EIM network and offering physical activity programming should be recorded on an ongoing basis.

## Implications for Public Health

With the increasing adoption of physical activity assessment, prescription, and referral by health care systems, there is a need to develop a comprehensive evaluation framework that clearly defines the types of evaluations necessary, key concepts to measure, and the steps involved with evaluation process. The evaluation framework described in this article is similar to other efforts that advance the evaluation of health disparities research (40), sustain community health initiatives (41), use health information systems and technology in complex health systems (42), and evaluate diabetes prevention and management initiatives (43). These evaluation approaches, much like ours, focus on a multitude of intervention outcomes, such as rates of participation and utilization of available resources. Our pragmatic evaluation framework accounts for individual and organizational factors related to patients receiving the appropriate level and type of physical activity assessment, counseling, prescription, and referral to supportive resources to reduce physical inactivity for the prevention and management of chronic health conditions.

Efforts to evaluate the EIM Solution in health care systems are likely to occur in real time alongside implementation efforts, rather than being carefully planned ahead of time (40). To prepare for this, we described a road map for evaluating the implementation of the EIM Solution in a health care system. Furthermore, many health clinics and systems may not have dedicated research or evaluation staff to plan a detailed evaluation plan or control timing of the implementation effort (44). Our fully defined framework can be used concurrently with implementation efforts to allow for an efficient evaluation process, while providing guidance on the roles and responsibilities of involved staff members.

A strength of our work originates from the pragmatic nature of this evaluation framework. Most of our recommendations can be executed with existing resources, independent of external or additional personnel. The use of the RE-AIM framework allows for the comparison of equivalent indicators across different health systems and clinics, providing greater generalizability of results when implementing the EIM Solution from one setting to the next. This framework also provides flexibility for cultural, contextual, and practical modifications in health settings. As health care leaders make choices about which components of the EIM Solution to implement in their health system, evaluators will be able to select the most relevant portions of this framework to develop a customized evaluation plan.

This evaluation framework is not intended to describe robust analytic approaches (ie, consideration of clustering, control of confounding factors and covariates) or strategies to ensure internal validity (ie, utilization of unbiased control groups). Instead, our intent is to outline strategies to capture system-level information on whether the EIM Solution is being implemented as intended using existing resources for data collection and analysis. Our evaluation framework provides the foundation for basic data collection that can be used in ongoing quality improvement efforts and as a part of future comprehensive analyses seeking to identify potential causal relationships. In health systems that adopt the EIM Solution, patient exposure (ie, quantity and quality) to the EIM Solution will likely vary. External evaluation teams may use various analytic approaches (ie, matched cohort studies, interrupted time series designs) to examine differences in health outcomes in patients receiving varying levels of care (or no care at all), as well as the potential impact of different covariates, just as they would for any other clinical information available in EMRs.

Although our framework provides guidance in evaluating the implementation of the EIM Solution in health systems, several challenges remain. Administrative hurdles and technological barriers, such as retrieving data from the EMR and accessing patient information and claims data (ie, ethical standards), may impede even the best-laid plans. Furthermore, some metrics valued by investigators for advancing the scientific field may not be as important to health care administrators, necessitating clear communication among involved parties in establishing a consensus on essential indicators to track. Whereas a research team may want to focus on characteristics of adoption to enhance scaling up in other health systems, administrators may be more interested in addressing low-performing providers or clinics and maximizing the return on their investment.

Even though we endeavored to develop a pragmatic evaluation model, the expanse of these recommendations can result in a complicated process if not carefully organized. We described several expanded metrics that investigators may want to consider if they have additional funding and resources when developing their evaluation plans. These expanded metrics might also serve as trouble-shooting mechanisms if the desired outcomes are not achieved. Lastly, one of the most important metrics may be the cost of implementing the EIM Solution in a health system across each of the RE-AIM indicators. Costs estimates, particularly as they relate to physical activity counseling and promotion, are not pragmatic measures regularly tracked or readily available in a health system. In determining the long-term value of the EIM Solution to health systems, these cost values must become a standardized, pragmatic measure.

We described a roadmap for assessing the implementation of the EIM Solution that can be used across a spectrum of health systems. As physical activity is increasingly integrated into health care systems, our pragmatic evaluation framework will be critical in determining the impact of the EIM Solution as a standard of care. This evaluation framework allows for the collection of data across multiple levels in a health system (patient, provider, and clinic settings) in a standardized format that can be compared with similar efforts taking place in other health settings. When using this framework, evaluation teams should ensure that the data being collected aligns with the mission of the health system and includes key metrics desired by clinicians and administrators to maximize the utility of the evaluation process for the health care system, and even more importantly, in support of improving patient health outcomes.
