# Erratum, Vol. 15, May 10 Release

**DOI:** 10.5888/pcd15.170344e

**Published:** 2018-05-31

Because of a technical error, Figure 1 and Figure 2 were switched in the HTML version of the article “A Pragmatic Application of the RE-AIM Framework for Evaluating the Implementation of Physical Activity as a Standard of Care in Health Systems.” The PDF version was correct as published. The article was corrected on May 14, 2018, and appears online at https://www.cdc.gov/pcd/issues/2018/17_0344.htm. We regret any confusion or inconvenience this error may have caused.

